# Supplementary material for: Plasma Sphingoid Base Profiles of Patients Diagnosed with Intrinsic or Idiosyncratic Drug-induced Liver Injury
Source: Int J Mol Sci. 2023 Feb 3;24(3):3013. doi: 10.3390/ijms24033013 (PMC9917723; doi:10.3390/ijms24033013)
Supplement: Supplementary file 1 [file ijms-24-03013-s001.zip › Table S2 and S3.pdf]

Table S2. Inclusion and Exclusion Criteria.

Inclusion criteria DILI	Exclusion criteria DILI and CONTROLS
➢ Age ≥ 18 years<br>➢ Maximum time between drug exposition and drug-related hepatotoxicity of 3 months<br>➢ Increased liver function parameters<br>➢ ALT or AST > 3x ULN or<br>➢ Total bilirubin> 2x ULN or<br>➢ ALP> 2x ULN<br>➢ Normal liver parameters according to last laboratory values before DILI onset (ALT, AST, Bilirubin, GGT, ALP)<br>➢ Rucam score ≥ 4	➢ Acute or chronic liver disease<br>➢ Cirrhosis (confirmed by sonography, radiology, histology, or documented in the patient's medical history)<br>➢ Acute viral hepatitis (hepatitis A, B, C, D, E, CMV, EBV, or HSV)<br>➢ Known chronic viral hepatitis B or C<br>➢ Alcoholic liver disease<br>➢ Non-alcoholic fatty liver disease or steatohepatitis<br>➢ Chronic storage disease (Wilson's disease, hemochromatosis or alpha-1 antitrypsin deficiency)<br>➢ Ongoing hepatic vascular disease (e.g. portal vein thrombosis, sinusoidal obstruction syndrome, Budd-Chiari syndrome)<br>➢ Ongoing or history of liver tumor or liver metastases<br>➢ Previous liver surgery (exception: cholecystectomy)
**Inclusion criteria CONTROLS**<br>➢ Age ≥ 18 years<br>➢ Normal liver function according to laboratory values ALT, AST, Bilirubin, GGT, and ALP	*See above*

ALP - alkaline phosphatase, ALT - alanine aminotransferase, AST - aspartate aminotransferase, GGT – gamma-glutamyl transferase, ULN - upper limit of normal

Table S3. DILI causitive agents.

Primary causitive agents	Patients [n]	Alternative suspected causitive agents	Patients [n]
**Amanita phalloides**	1		
**acetaminophen (intoxication)**	7		
**acetazolamide, topiramate**	1		
**amiodarone**	1	acetaminophen, metamizole	1
**amoxicillin/clavulanic acid**	4	acetaminophen, metamizole	1
**atorvastatin**	4	dalteparin	1
		pravastatin, acetaminophen, metamizole	1
		etorixib	1
		acetominphen	1
**cetriaxone**	3	rifampicin/pyrazinamide/isoniazid/ethambutol, fluconazole, dalteparin	1
		metamizole	1
		clarithromycin, flucloxacillin, amoxicillin/clavulanic acid, gentamicin, levofloxacin	1
**pentamidine**	1	cetriaxone	1
**piperacillin/tazobactam**	2	vancomycin, amoxicillin/clavulanic acid, meropenem, teicoplanin, acetaminophen	1
**rifampicin/pyrazinamide/isoniazid**	1		
**sulfamethoxazole/trimethoprim**	1		
**tacrolimus/mycophenolate**	1	amoxicillin/clavulanic acid	1
**trimipramine**	1	etoricoxib, metamizole	1
	28		13

Cases of patients recruited between 2012-2015 were reviewed by a panel of independent experienced clinicians, including at least one clinical pharmacologist. For adjudication to the DILI cohort, a consensus agreement of the majority of the panel was required. The RUCAM score was used in the assessment of causality by the panel, whereby the scoring was conducted for several candidate causative agents, applied separately to individual drugs and drug combinations as listed within the table. When more than one drug or drug combination was rated “possible” or higher by RUCAM, they were ranked in order of likelihood of causing DILI (signature pattern of DILI, literature, incidence), leading to the identification of the primary and alterative causative agents listed within the table [59, 60].

Bibliography

59. Church, R. J.; Kullak-Ublick, G. A.; Aubrecht, J.; Bonkovsky, H. L.; Chalasani, N.; Fontana, R. J.; Goepfert, J. C.; Hackman, F.; King, N. M. P.; Kirby, S.; Kirby, P.; Marcinak, J.; Ormarsdottir, S.; Schomaker, S. J.; Schuppe-Koistinen, I.; Wolenski, F.; Arber, N.; Merz, M.; Sauer, J. M.; Andrade, R. J.; van Bommel, F.; Poynard, T.; Watkins, P. B., Candidate biomarkers for the diagnosis and prognosis of drug-induced liver injury: An international collaborative effort. *Hepatology* **2019,** 69, (2), 760-773.

60. Aithal, G. P.; Watkins, P. B.; Andrade, R. J.; Larrey, D.; Molokhia, M.; Takikawa, H.; Hunt, C. M.; Wilke, R. A.; Avigan, M.; Kaplowitz, N.; Bjornsson, E.; Daly, A. K., Case definition and phenotype standardization in drug-induced liver injury. *Clinical pharmacology and therapeutics* **2011,** 89, (6), 806-15.
